# Robust Shape-from-Focus via Physics-Inspired Distortion-Aware Focal Depth Regression

**DOI:** 10.3390/s26113390

**Published:** 2026-05-27

**Authors:** Xin Li, Wei Shen, Jian Li, Zhongsheng Zhai, Xuhong Guan, Zili Lei

**Affiliations:** 1 Hubei Key Laboratory of Modern Manufacturing Quantity Engineering, School of Mechanical Engineering, Hubei University of Technology, Wuhan 430068, China; 18171870097@163.com (X.L.); 102400017@hbut.edu.cn (W.S.); zs.zhai@hbut.edu.cn (Z.Z.); 15327958028@163.com (X.G.); 2 School of Mechanical and Electrical Engineering, Changsha University, Changsha 410022, China; z20190620@ccsu.edu.cn

**Keywords:** Shape-from-Focus, high dynamic range, weak-textured surfaces, physics-guided depth regression, channel-wise feature attention, confidence-guided smoothing

## Abstract

Shape-from-Focus (SFF) is attractive for microscopic three-dimensional measurement, but high dynamic range (HDR) surfaces and weak-textured surfaces distort the focus curve through saturation, spurious peaks, and low signal-to-noise ratios. These distortions violate the unimodal assumption used by Gaussian peak localization and limit post-processing-only correction. This paper proposes a physics-guided distortion-aware SFF pipeline for opaque single-surface targets. The Distortion-Aware Focal Depth Regression Network (DAFDR-Net) learns from synthetic focus-curve distortions and uses Channel-wise Feature Attention (CFA) and Soft Peak Localization to reweight distortion-sensitive temporal-response features while preserving a peak-localization prior. Its foreground validity output is further used for confidence-guided adaptive smoothing. On an HDR free-form surface dataset, the proposed pipeline reduces RMSE by 36.5% relative to an MRF optimization method and compresses the 99th-percentile absolute error from 0.181 to 0.033. On weak-textured monocrystalline silicon wafer data, it reduces flat-region depth standard deviation by 51.3%.

## 1. Introduction

### 1.1. Background and Motivation

Three-dimensional surface measurement techniques have extensive applications in aerospace, intelligent manufacturing, medical devices, and cultural heritage preservation [1,2]. Shape-from-Focus (SFF) is a class of passive optical measurement techniques that has been widely adopted in microscopic measurement scenarios [3,4] due to its advantages of low cost, flexible deployment, and insensitivity to occlusion [5,6,7].

The physical foundation of SFF originates from the thin lens imaging model: when an object point lies on the focal plane, its image point is sharply focused; when it deviates from the focal plane, a circle of confusion forms and the image becomes blurred [8]. By acquiring an image sequence at different focal positions along the optical axis, measuring the degree of focus for each frame using a Focus Measure Operator (FMO), and then determining the optimal focus position for each pixel through peak localization, the three-dimensional surface topography of the object can be recovered [9,10].

In industrial practice, SFF faces two typical challenging scenarios—high dynamic range (HDR) surfaces and weak-textured surfaces. HDR surfaces such as precision metal components and mechanical watch movements exhibit coexisting specular highlights and shadow regions, making it difficult for the sensor’s dynamic range to fully capture focus information [11]. Weak-textured surfaces such as monocrystalline silicon wafers and optical elements lack significant grayscale variations, resulting in extremely low signal-to-noise ratios in the FMO response [12]. Both scenarios induce severe distortions in focus curves, including saturation truncation, ghost peaks (spurious peaks), and baseline elevation, causing traditional peak localization methods to fail.

### 1.2. Limitations of Existing Methods

Existing SFF improvement efforts can be categorized into three aspects: focus measure operators, depth estimation methods, and depth map post-processing.

**Focus Measure Operators.** Various operators have been proposed in the literature, including Laplacian [13], Tenenbaum gradient [14], gray-level variance [15], and wavelet energy [16] operators. Pertuz et al. [17] conducted a systematic comparison of 36 operators. The effectiveness of operator improvements is limited to enhancing the signal-to-noise ratio of focus curves without fundamentally addressing the curve distortion problems in HDR and weak-texture scenarios.

**Depth Estimation Methods.** Traditional methods localize focus curve peaks using discrete maximum search or Gaussian interpolation [8,18], with the underlying assumption that the curve exhibits a symmetric unimodal shape. This assumption is frequently violated in HDR and weak-texture scenarios (see Section 2.2 for details). Deep learning methods have recently been introduced to the SFF domain [19], but existing networks are trained on data that does not encompass actual distortion patterns, limiting their robustness.

**Depth Map Post-processing.** Common methods are divided into local filtering (Gaussian filtering, bilateral filtering, guided filtering, etc.) [20,21] and global optimization (such as Markov Random Field, MRF) [22,23,24]. He et al. [11] adopted an MRF energy minimization framework to introduce spatial consistency constraints. Existing post-processing methods have two limitations: first, they do not differentiate pixel-wise confidence, allowing erroneous observations from low-confidence pixels to be retained; second, post-processing is essentially a remedial measure for errors that have already occurred, and its effectiveness is limited when upstream depth estimation fails extensively.

### 1.3. Contributions

This paper targets a specific metrological problem: recovering the single-surface depth of opaque objects from SFF focus curves that are distorted by HDR saturation, specular interference, or weak-texture noise. The proposed method differs from focus-measure redesign because it not only enhances the input curve, but it also learns to localize the physically plausible peak from distorted curves. It also differs from MRF-style post-processing because confidence is estimated before spatial smoothing, reducing the number of erroneous pixels that must be repaired after the fact. Compared with existing learning-based SFF regressors trained on ideal Gaussian-like curves, DAFDR-Net is trained on physically motivated distortion classes and embeds peak-localization and channel-wise feature-attention priors in a lightweight one-dimensional architecture.

The main contributions are as follows.

1. **Source-level distortion-aware depth regression.** We construct synthetic focus-curve distortions that represent saturation truncation, ghost peaks, low-SNR weak peaks, asymmetry, and background noise, enabling DAFDR-Net to estimate depth directly from hard curves rather than relying only on post-processing.
2. **Physics-guided and interpretable network design.** Kernel sizes are tied to the depth-of-field-to-step ratio, the CFA module reweights temporal-response feature channels associated with distorted focus-curve patterns, and SPL provides a differentiable single-peak localization prior for opaque single-surface targets.
3. **Confidence-guided spatial refinement and validation.** The network outputs both depth and validity probability, allowing adaptive smoothing to preserve reliable structures while replacing low-confidence pixels. Experiments on HDR simulated data and weak-textured silicon wafer data show consistent reductions in bulk error, tail error, and flat-region roughness.

### 1.4. Paper Organization

The remainder of this paper is organized as follows: Section 2 reviews related work on focus measure operators, depth estimation methods, and depth map optimization. Section 3 presents the distortion-aware depth regression network and confidence-guided adaptive smoothing method. Section 4 validates the proposed method through simulation and real-world experiments. Section 5 concludes the paper and discusses future research directions.

## 2. Related Work

### 2.1. Focus Measure Operators

SFF acquires a stack of *K* images along the optical axis to estimate the surface depth of an object. Let the aligned grayscale sequence be ${\left\{I_{k}\left(x,y\right)\right\}}_{k=1}^{K}$. The focus measure $FM_{k}\left(p\right)=\Phi \left(I_{k},U\left(p\right)\right)$ is computed over a w×w neighborhood U(p) of pixel *p*, yielding the focus curve $f_{p}\left(k\right)=FM_{k}\left(p\right)$.

Under ideal imaging conditions, $f_{p}\left(k\right)$ exhibits a unimodal shape near the best focus position and approximately follows a Gaussian distribution. However, low signal-to-noise ratios in weak-textured regions and saturation with specular diffusion caused by HDR surfaces lead to curve distortions (baseline elevation, peak broadening, and spurious peak interference; see Section 2.2 for details), thereby degrading the stability of depth recovery.

Various focus measure operators have been proposed in the literature: Laplacian-based second derivative operators [13], Tenenbaum gradient operators [14], gray-level variance operators [15], and wavelet energy operators [16]. Pertuz et al. [17] conducted a systematic comparison of 36 operators. Classical focus evaluation functions based on correlation and statistics also exist in the autofocus domain [25,26]. These operators characterize the degree of focus from the perspectives of spatial differentiation, statistical properties, and frequency-domain energy. Spatial derivative and statistical operators are susceptible to noise dominance in weak-textured regions, while frequency-domain ratio operators may amplify errors under exposure variations.

### 2.2. Depth Estimation Methods

**Figure 1:**
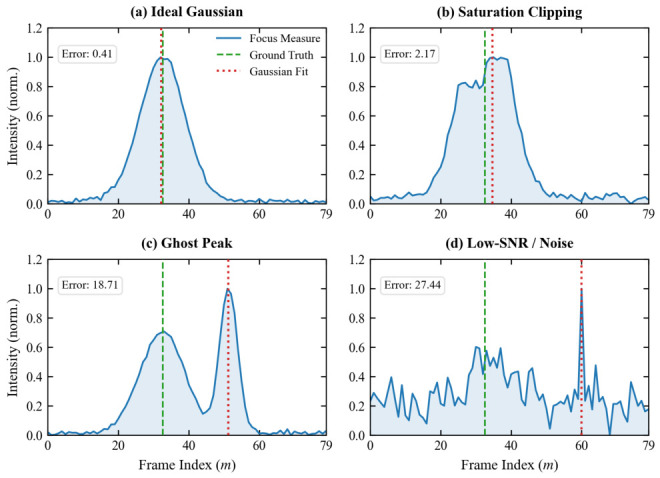
Performance of traditional Gaussian interpolation under different scenarios: (**a**) ideal Gaussian curve with interpolation error of approximately 0.65 frames; (**b**) saturation truncation causes an asymmetric curve with error increasing to 6.13 frames; (**c**) ghost peak leads to peak misjudgment with error reaching 19.72 frames; (**d**) weak-texture low SNR causes noise peak misjudgment, with error reaching 29.33 frames. Green dashed lines indicate ground truth depth positions; red dotted lines show Gaussian interpolation results.

Once the per-pixel focus curve $f_{p}\left(k\right)$ is obtained, the core task of depth estimation is to localize its peak. Discrete maximum search is limited by the scanning step size, while Gaussian interpolation improves sub-frame precision by fitting the neighborhood of the discrete maximum [5,8,18]. The detailed baseline equations are provided in the Supplementary Materials. Gaussian interpolation assumes that the focus curve exhibits a symmetric unimodal shape, which can improve depth estimation accuracy under ideal imaging conditions. However, this assumption is frequently violated in HDR and weak-textured surface measurements [11,12,27]. Figure 1 illustrates three typical distortion patterns in challenging scenarios: (1) **saturation truncation**—insufficient sensor dynamic range causes asymmetric flattening of the curve peak; (2) **ghost peaks**—specular reflection or FM window crossing depth discontinuities produces spurious peaks; (3) **weak-texture low SNR**—weak focus response causes noise peaks to be misjudged as true peaks.

Deep learning methods have recently been introduced to the focal stack depth estimation domain. Yang et al. [28], Won et al. [29], and Kang et al. [30] proposed depth estimation networks for natural scenes that directly regress depth maps from image sequences. These methods rely on large-scale real annotated datasets for training, have relatively heavy network architectures, and are designed for indoor/outdoor natural scenes, making them difficult to directly apply to industrial microscopic measurement. In the microscopic measurement direction, Mutahira et al. [19] proposed a focus curve depth regression method based on lightweight fully connected networks, directly mapping normalized FM curves to depth values. However, their training data only includes ideal Gaussian curves with mild noise without covering typical industrial scenario distortions such as HDR saturation truncation, ghost peaks, and weak-texture low SNR, limiting robustness in actual challenging surface measurements.

### 2.3. Depth Map Optimization Methods

The initial depth map obtained from focus curve peak localization often contains noise and outliers. Existing post-processing methods are divided into local filtering and global optimization approaches. Local filtering methods (bilateral filtering [31], guided filtering [32], etc.) are computationally simple but prone to over-smoothing [20]. Global optimization methods such as MRF introduce spatial consistency through energy minimization [22,23,24,33]; a representative formulation is summarized in the Supplementary Materials. MRF can achieve globally consistent depth optimization, but existing methods still have two limitations: the data term applies equal weight to all pixels, allowing erroneous observations from low-confidence pixels to be retained; furthermore, focus curve quality is not explicitly utilized to adjust the optimization strength.

### 2.4. Conceptual Comparison with Existing Methods

To clarify how the proposed pipeline differs from existing categories of SFF methods rather than merely combining existing modules, Table 1 contrasts five representative method families along the axes that matter for distorted focus curves: how curve distortions are handled, whether per-pixel confidence is estimated, whether spatial consistency is enforced, the underlying architecture, and the training-data assumption. The central distinction is that DAFDR-Net handles distortion upstreamat the depth-regression stage and exposes an explicit per-pixel validity score that closes the loop with confidence-guided smoothing, whereas existing approaches either assume an undistorted unimodal curve or attempt to repair errors only after they have propagated into the depth map.

## 3. Method

### 3.1. Focus Feature Extraction

The proposed method follows a three-stage pipeline: “focus feature extraction—distortion-aware depth regression—confidence-guided post-processing”. A compact processing diagram is provided in the Supplementary Materials, and this section presents each stage in sequence.

The defocusing process can be approximated as the convolution of the in-focus image with the point spread function, where high-frequency details attenuate as the degree of defocus increases. This paper employs two-dimensional discrete wavelet transform to extract the first-level detail subband energy as the focus measure [16,34,35]. To suppress the cumulative baseline elevation caused by noise coefficients under weak-texture and HDR interference, a threshold-based filtering is introduced for the detail energy, retaining only significant coefficients for accumulation:

(1) $$FM^{\left(k\right)}\left(p\right)=\sum_{\left(x,y)\in U(p\right)}\sum_{s\in \{LH_{1},HL_{1},HH_{1}\}}{\left(W_{s}^{\left(k\right)}\left(x,y\right)\right)}^{2}\;I\;\left({\left(W_{s}^{\left(k\right)}\left(x,y\right)\right)}^{2}>T\right),$$

where $W_{s}^{\left(k\right)}$ denotes the detail subband coefficients of the *k*-th frame after wavelet decomposition ($LH_{1}$, $HL_{1}$, and $HH_{1}$ correspond to horizontal, vertical, and diagonal directions, respectively), U(p) is the w×w neighborhood of pixel *p*, I(·) is the indicator function, and *T* is the threshold. This strategy effectively suppresses the cumulative influence of small noise coefficients while preserving significant energy responses at edges and textures in focused frames.

The focus measure maps are computed frame by frame and stacked to form the focus volume F(x,y,k). The focus curve $f_{p}\left(k\right)=F\left(p,k\right)$ is extracted per pixel. For network processing and cross-pixel comparison, the focus curve is normalized by its maximum value:

(2) $${\tilde{f}}_{p}\left(k\right)=\frac{f_{p}\left(k\right)}{\max_{1\leq k\leq K}f_{p}\left(k\right)+\varepsilon },$$

where ε is a small constant to prevent division by zero. The normalized focus curve ${\tilde{f}}_{p}\left(k\right)$ serves as the input to the depth regression network.

### 3.2. Physics-Guided Distortion-Aware Depth Regression Network

Traditional peak localization frequently fails under HDR and weak-texture conditions. This paper proposes the physics-guided Distortion-Aware Focal Depth Regression Network (DAFDR-Net), targeting opaque surfaces with a single physical depth per pixel [36]. Its design follows a hybrid paradigm of “physics-prior guidance with data-driven optimization”: convolutional layers encode local geometric features of focus curves; the Channel-wise Feature Attention (CFA) module reweights temporally encoded feature channels that are sensitive to distorted focus-curve patterns; the Soft Peak Localization (SPL) mechanism provides geometric inductive bias for single-surface depth regression; and the multi-task learning framework leverages the intrinsic correlation between depth and validity for joint optimization [37]. This design enables the network not only to localize true peak positions from distorted curves but also to maintain interpretability with respect to the underlying physical process.

#### 3.2.1. Distortion-Aware Training Data

**Figure 2:**
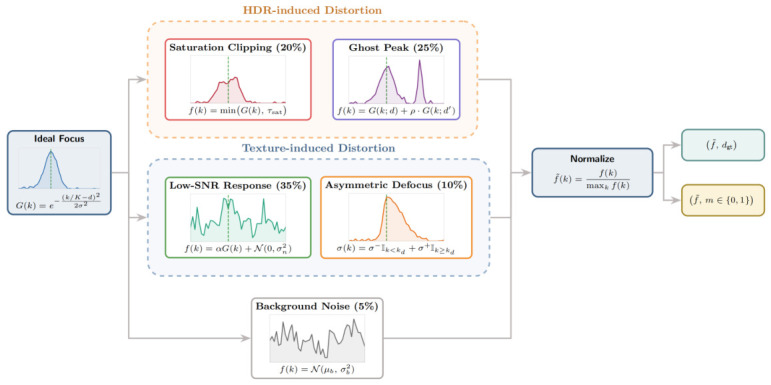
Distortion-aware training data generation pipeline and synthetic FM curve examples. Green dashed lines indicate the ground truth depth position $d_{\mathrm{true}}$.

Existing deep learning SFF methods typically train on data covering only ideal unimodal curves [19], making it difficult to handle complex distortions encountered in practical measurements. This paper proposes a synthetic training data generation strategy encompassing multiple typical distortion patterns (Figure 2), including, saturation (20%), ghost peak (25%), low-SNR (35%), asymmetric (10%), and background noise (5%).

Distortion transformations are applied to an ideal Gaussian curve as the base element:

(3) $$G\left(k;d,\sigma \right)=\exp\left(-\frac{{\left(k/K-d\right)}^{2}}{2\sigma^{2}}\right),\;k=1,\ldots ,K,$$

where d∈(0,1) is the normalized ground truth depth and σ is the standard deviation controlling peak width. The synthesis methods for each distortion type are as follows:

- **Saturation truncation**: $f\left(k\right)=\min\left(G\left(k\right),\tau_{\mathrm{sat}}\right)$, where $\tau_{\mathrm{sat}}\in \left[0.6,0.9\right]$ is the saturation threshold, simulating peak flattening caused by insufficient sensor dynamic range. This per-curve model captures the temporal saturation plateau but not the lateral electron-overflow (blooming) effect [38]; in practice, lateral blooming manifests as spatially correlated plateau widening. The FM operator (computed over a 9×9 neighborhood) and the CFA-based feature reweighting (Section 3.2.2) may reduce sensitivity to some curve-level artifacts, but they do not explicitly correct spatial blooming. A more explicit lateral blooming model is left as future work (Section 4.4).
- **Ghost peak**: $f\left(k\right)=G\left(k;d,\sigma \right)+\rho \cdot G\left(k;d^{\prime },\sigma^{\prime }\right)$, where $d^{\prime }=d+\Delta d$ is the spurious peak position (Δd randomly sampled), and ρ∈[0.3,1.2] is the relative amplitude of the spurious peak, simulating secondary peaks caused by specular reflection or FM window crossing depth discontinuities.
- **Low-SNR weak peak**: f(k)=α·G(k)+n(k), where α∈[0.05,0.3] is the weak-texture attenuation coefficient and $n\left(k\right)∼N\left(0,\sigma_{n}^{2}\right)$ is additive noise, simulating low signal-to-noise ratio responses in weak-textured regions.
- **Asymmetric**: Piecewise standard deviation $\sigma \left(k\right)=\sigma^{-}I\left(k<k_{d}\right)+\sigma^{+}I\left(k\geq k_{d}\right)$ with $\sigma^{-}\neq \sigma^{+}$, simulating peak skewness caused by directional differences in the defocusing process.
- **Background noise**: $f\left(k\right)∼N\left(\mu_{b},\sigma_{b}^{2}\right)$, pure noise sequences without valid peaks, serving as negative samples for background regions.

The above distortion parameter ranges (e.g., $\tau_{\mathrm{sat}}\in \left[0.6,0.9\right]$, ρ∈[0.3,1.2], α∈[0.05,0.3]) are determined based on observations of typical distorted curves in real HDR and weak-textured data, covering common distortion intensity intervals in industrial measurement scenarios. Hard samples (with distortions) constitute approximately 90%, while 5% ideal Gaussian curves are retained to ensure basic fitting capability for normal samples. The data generation strategy can be parameterized according to the sequence length *K* of the target dataset: given *K*, synthetic training samples of corresponding length are generated according to the above proportions and parameter ranges, and the network is trained accordingly. Due to significant differences in sequence lengths across different acquisition configurations, this paper generates training data and trains independent models for each sequence length rather than adopting a single universal model.

To verify that the proposed proportions do not overfit to the test distribution, a sensitivity analysis was performed by perturbing the class proportions and retraining; the full sweep is reported in the Supplementary Materials. Within ±10–15 percentage-point shifts of the four hard-sample classes, RMSE on the HDR test set varies by less than ∼8% relative (from 0.015 to 0.016), and a uniform proportion (each class ≈17%) yields RMSE 0.016 (+5% relative). Only severe imbalance, such as inflating the saturation class to 50% (+30 percentage points), causes a noticeable degradation (+19% relative). The strategy is therefore robust to moderate proportion changes and is not overfitted to the specific test set.

The transferability of this synthesis strategy stems from the physical interpretability of the distortion categories: saturation truncation originates from sensor dynamic range limitations; ghost peaks arise from FM windows crossing depth boundaries or specular reflection; weak-peak noise results from weak focus response due to texture absence. These mechanisms can appear across acquisition systems and measured surfaces, but their quantitative distributions are configuration- and sensor-dependent; therefore, the synthesis parameters and trained weights should be revalidated or regenerated when the optical system or sensor type changes.

#### 3.2.2. Network Architecture

**Figure 3:**
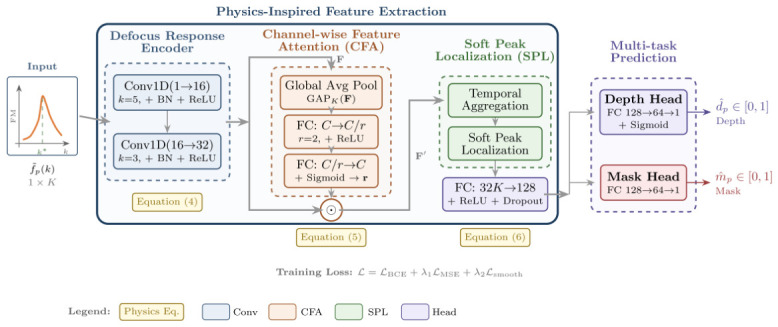
Architecture of the physics-guided Distortion-Aware Focal Depth Regression Network (DAFDR-Net). The network comprises a defocus response encoding module, Channel-wise Feature Attention (CFA) module, Soft Peak Localization (SPL) mechanism, and multi-task prediction heads. Input: normalized focus sequence ${\tilde{f}}_{p}\left(k\right)$; Output: depth estimate ${\hat{d}}_{p}$ and foreground mask probability ${\hat{m}}_{p}$. The CFA weights r reweight temporal-response feature channels, while soft peak localization provides geometric inductive bias.

The overall architecture of DAFDR-Net is shown in Figure 3, comprising four components: defocus response encoding module, Channel-wise Feature Attention (CFA) module, Soft Peak Localization (SPL) mechanism, and multi-task prediction heads.

**Defocus Response Encoding Module.** This module employs two layers of 1D-CNN to extract local geometric features of focus curves. The selection of convolution kernel sizes is based on scale analysis of the defocus physical model: in thin lens imaging, the diameter of the circle of confusion is approximately linearly related to the defocus amount [8], and the effective response interval of the focus curve typically spans several to a dozen frames. The kernel sizes are therefore determined by the physical depth-of-field-to-step ratio rather than chosen as a global constant:

(4) $$k_{\mathrm{coarse}}\approx \left\lceil \frac{\sigma_{\mathrm{DoF}}}{\Delta z}\right\rceil ,\;k_{\mathrm{fine}}\approx \left\lceil \frac{k_{\mathrm{coarse}}}{2}\right\rceil ,$$

where $\sigma_{\mathrm{DoF}}$ is the physical depth-of-field along the optical axis and Δz is the scanning step size. For the two acquisition configurations used in this work, the corresponding $\sigma_{\mathrm{DoF}}/\Delta z$ ratios both fall near 5: in the free-form HDR setup (Δz=20μm, simulated objective with $\sigma_{\mathrm{DoF}}\approx 100\;\mu m$) and in the silicon-wafer microscope setup (high-NA objective with $\sigma_{\mathrm{DoF}}\approx 0.5\;\mu m$, Δz≈0.1μm). Equation (4) therefore yields $k_{1}=5$ and $k_{2}=3$ for both configurations. For a different objective with a different $\sigma_{\mathrm{DoF}}/\Delta z$ ratio, kernel sizes would be recomputed via Equation (4) and the network retrained with parametrically regenerated synthetic data of matching sequence length, as documented in Section 3.2.1. Adaptive alternatives such as dilated convolutions [39] and deformable/dynamic kernels [40] could enable a single model to span multiple DoF regimes at the cost of additional parameters; this trade-off is discussed in Section 4.4. To verify that this choice is not merely empirical, we performed kernel-size and depth sensitivity sweeps on the HDR free-form surface dataset (full tables provided in the Supplementary Materials). Kernel pairs smaller than $\left\lceil \sigma_{\mathrm{DoF}}/\Delta z\right\rceil$ (e.g., $k_{1}\;=\;3,k_{2}\;=\;3$, RMSE 0.019) under-cover the defocus response, while larger kernels (e.g., $k_{1}\;=\;9,k_{2}\;=\;5$, RMSE 0.020) over-smooth fine peak features; the physics-keyed choice $\left(k_{1},k_{2}\right)\;=\;\left(5,3\right)$ achieves the lowest RMSE of 0.015. Increasing depth beyond 2 layers does not improve accuracy: 3 and 4 layers give RMSE values of 0.015 and 0.016, respectively, while also increasing the parameter count. Therefore, the 2-layer baseline is retained for efficiency. Batch Normalization (BN) follows the convolutional blocks to stabilize training [41].

**Channel-wise Feature Attention (CFA) Module.** In ideal defocus imaging, the FM response near the focused frame follows an approximately Gaussian distribution, and responses far from the focused frame should decay monotonically. However, distorted curves (saturation, ghost peaks, noise-dominated) violate this prior and produce unstable temporal-response feature patterns. This paper therefore introduces a channel-wise feature attention module, implemented in the squeeze-and-excitation style [42], to learn weights for temporally encoded feature channels rather than explicit per-frame reliability weights:

(5) $$r=\sigma \left(W_{2}\cdot \delta \left(W_{1}\cdot {\mathrm{GAP}}_{K}\left(F\right)\right)\right),\;F_{c,k}^{\prime }=r_{c}F_{c,k},$$

where $F\in R^{C\times K}$ is the convolutional feature, ${\mathrm{GAP}}_{K}\left(\cdot \right)$ denotes global average pooling over the temporal/frame dimension, $r\in R^{C}$ is the channel-wise attention vector, $W_{1}\in R^{(C/r)\times C}$ and $W_{2}\in R^{C\times (C/r)}$ are learnable parameters (compression ratio r=2), δ(·) is ReLU activation, and σ(·) is Sigmoid activation. Because the temporal index is pooled before generating r, CFA should be interpreted as channel-wise attention over temporal-response features, not as a direct estimator of individual frame reliability $r_{k}$. It suppresses feature channels that tend to respond strongly to saturation, ghost peaks, or noise-dominated curves, thereby indirectly reducing the influence of distorted temporal responses in the subsequent depth regression.

*Failure safeguard.* On ultra-smooth surfaces whose surface texture lies near the sensor quantization-noise floor [43], the CFA module alone could in principle amplify noise-sensitive feature channels as if they carried defocus information. The multi-task validity head ${\hat{m}}_{p}$ is the explicit safeguard: the **Background-noise** synthetic class (Section 3.2.1, 5% of training samples) trains the network to map curves dominated by quantization-level noise to ${\hat{m}}_{p}\to 0$ rather than to a fabricated depth, while the confidence-guided smoothing (Section 3.3) replaces such pixels by neighborhood interpolation. This mechanism is consistent with the silicon-wafer results, where the smoothed output further reduces the flat-region standard deviation relative to the direct DAFDR-Net output.

**Soft Peak Localization (SPL) Mechanism.** Traditional Gaussian interpolation performs analytical fitting on three points in the discrete peak neighborhood. This paper introduces differentiable soft peak localization in the network as an inductive bias. Let the CFA-weighted feature $F^{\prime }$ yield a sequence-level representation $h\in R^{K}$ after pooling. The soft peak position can be expressed as

(6) $${\hat{k}}_{\mathrm{soft}}=\sum_{k=1}^{K}k\cdot {\mathrm{softmax}}_{k}\left(h\right).$$

This mechanism provides a geometric prior for “peak localization” to the network, making depth regression a differentiable approximation inspired by physical processes rather than a pure black-box mapping. In practice, soft peak information is implicitly encoded in the depth prediction through fully connected layers.

**Multi-task Prediction Heads.** Shared features branch into depth regression and validity discrimination heads through fully connected layers, outputting normalized depth ${\hat{d}}_{p}$ and foreground mask probability ${\hat{m}}_{p}$ (during inference, the binary mask is obtained as $m_{p}=I\left({\hat{m}}_{p}>0.5\right)$), respectively. Dropout follows the fully connected layers to prevent overfitting [44]. This design follows the shared representation paradigm of multi-task learning [37], where the complementarity between depth estimation and validity discrimination tasks facilitates learning more robust feature representations.

The detailed layer-by-layer parameter summary is provided in the Supplementary Materials. For K=44, the network has approximately 200k parameters; for longer sequences, the parameter count grows linearly in the aggregation layer.

#### 3.2.3. Training Objective

Joint optimization of depth regression and mask discrimination is employed. The loss function design balances task objectives and physical constraints:

(7) $$L=L_{\mathrm{BCE}}+\lambda_{1}L_{\mathrm{MSE}}+\lambda_{2}L_{\mathrm{smooth}},$$

where $L_{\mathrm{BCE}}$ is the binary cross-entropy loss for validity discrimination, $L_{\mathrm{MSE}}$ is computed only on foreground depth samples, and $L_{\mathrm{smooth}}$ is an auxiliary smoothness regularization. In experiments, $\lambda_{1}=10$ and $\lambda_{2}=0.01$ are used to prioritize depth accuracy while keeping the auxiliary regularization mild. Detailed term definitions are given in the Supplementary Materials.

#### 3.2.4. Validation on Synthetic Data

To validate the effectiveness of the distortion-aware training strategy before full-image reconstruction, a preliminary synthetic-curve test is conducted with non-overlapping training and test samples. For K=44, DAFDR-Net reduces hard-sample MAE from 0.055 for Gaussian interpolation to 0.021, with 100% foreground/background mask accuracy. The complete synthetic validation table is reported in the Supplementary Materials, while full-image reconstruction results are presented in Section 4.

### 3.3. Confidence-Guided Adaptive Smoothing

The per-pixel regression of DAFDR-Net still introduces spatial noise and a small number of outliers. To incorporate spatial consistency with low computational overhead, this paper proposes confidence-guided adaptive smoothing: pixel-wise confidence is used to modulate bilateral filtering strength [31,45], combined with outlier correction, achieving a balance between detail preservation in high-confidence regions and noise suppression in low-confidence regions.

#### 3.3.1. Confidence Construction

The foreground mask probability ${\hat{m}}_{p}$ output by the network serves as a prior, which is fused with a focus curve quality factor. This paper characterizes the curve signal-to-noise ratio using the peak sharpness of the normalized focus curve ${\tilde{f}}_{p}\left(k\right)$ [46]:

(8) $$S\left(p\right)=\frac{f_{\max}\left(p\right)-f_{\mathrm{mean}}\left(p\right)}{f_{\max}\left(p\right)+\epsilon },$$

where $f_{\max}\left(p\right)=\max_{k}{\tilde{f}}_{p}\left(k\right)$, $f_{\mathrm{mean}}\left(p\right)$ is the curve mean, and ϵ is a small constant to prevent division by zero. The final confidence is defined as

(9) $$C\left(p\right)={\hat{m}}_{p}\cdot S\left(p\right).$$

When a pixel belongs to a valid region and the curve peak is distinct, C(p) is high; otherwise, it decreases to enhance subsequent smoothing. In implementation, $C\left(p\right)={\hat{m}}_{p}$ can also be used directly for simplification.

#### 3.3.2. Adaptive Bilateral Filtering

The filtering strength is dynamically adjusted according to pixel confidence using an adaptive bilateral filter [31,47,48]. The spatial scale increases in low-confidence regions for stronger denoising and remains smaller in high-confidence regions to preserve edges and reliable surface details. The depth-range term is kept fixed to protect true discontinuities. Detailed filtering equations and the full processing diagram are provided in the Supplementary Materials.

#### 3.3.3. Outlier Correction

To suppress isolated outliers, local depth-gradient anomalies are detected with a three-sigma statistical threshold [49]. Flagged pixels are then replaced by a confidence-weighted average of non-outlier neighbors, prioritizing depth information from pixels with reliable focus curves. The explicit outlier equations are moved to the Supplementary Materials to keep the main method focused on the core mechanism.

## 4. Experiments and Analysis

### 4.1. Experimental Setup

This section validates the proposed method on two datasets: the publicly available HDR free-form surface simulation dataset by He et al. [11] and the monocrystalline silicon wafer real-world dataset collected in this work.

**Simulated Free-form Surface Dataset.** This dataset was rendered using Blender 3.0.0 (Blender Foundation, Amsterdam, The Netherlands), with the test object being a stone block with undulating surfaces and deep grooves. The image resolution is 2000×2000 pixels, and the focal plane scanning range is 0.02–0.88 mm with a step size of 0.02 mm, yielding a 44-frame sequence. Ground truth depth maps are directly exported from the rendering engine. This dataset exhibits typical HDR characteristics, prone to saturation truncation and ghost peak distortions in focus curves, making it suitable for testing the robustness of depth estimation methods in challenging scenarios.

**Real-world Dataset (Monocrystalline Silicon Wafer).** To validate the applicability of the proposed method in real industrial scenarios, multi-focus image sequences of monocrystalline silicon wafer samples were collected using a variable-focus surface topography measurement system comprising a microscope objective, precision motorized translation stage, industrial camera, and ring LED illumination. The wafer exhibits a two-layer depth structure with extremely weak surface texture; the mesa edge regions have extremely low signal-to-noise ratios and are prone to spike-type outlier depths. The precision stage controls the objective to scan along the optical axis at uniform step intervals, acquiring a 3500-frame image sequence at 640×480 pixel resolution. The system photograph and representative raw frames are provided in the Supplementary Materials.

**Evaluation Metrics.** For the simulated dataset, Root Mean Square Error (RMSE) and Mean Absolute Error (MAE) are computed on the valid ground-truth mask, consistent with the original literature. To characterize the simulated error distribution beyond bulk RMSE/MAE, we additionally report 95th- and 99th-percentile absolute errors, maximum absolute error, and error standard deviation. For the silicon-wafer dataset, internal flat-region consistency is evaluated using plateau standard deviations, and the extended plateau statistics include the ISO 25178 areal roughness parameters $S_{a}$ and $S_{q}$ [50], together with residual skewness and kurtosis. Metric definitions and supplementary statistics are provided in the Supplementary Materials.

**Implementation Details.** The proposed method is implemented using the PyTorch (Version 2.0.1) framework. The sequence lengths for the simulation dataset and silicon wafer dataset are K=44 and K=3500, respectively; both use networks with different input lengths and training data, with model parameters not shared. The main parameter settings are as follows:

- **Focus measure operator**: Wavelet energy operator (Equation (1)), window size 9×9 [34,35];
- **Network architecture**: DAFDR-Net (defocus response encoding + CFA + multi-task heads), ReLU activation, Dropout 0.3 [44];
- **Training configuration**: Adam optimizer [51], learning rate $10^{-4}$, weight decay $10^{-4}$, batch size 128, maximum 100 epochs; ReduceLROnPlateau (patience = 7, factor = 0.5) and early stopping (patience = 15); loss weights $\lambda_{1}=10$, $\lambda_{2}=0.01$ (see Section 3.2.3);
- **Other settings**: 10,000 synthetic FM curve samples (see Section 3.2.1; train/val/test split detailed below); adaptive smoothing window 21×21, $\sigma_{\mathrm{base}}=3$, $\sigma_{r}=0.03$, γ=2.

**Dataset Partitioning.** The 10,000 synthetic focus-curve samples are generated parametrically with disjoint random seeds and split into a 70% training set (7000 curves), 15% validation set (1500), and 15% test set (1500). The class proportions in Section 3.2.1 are preserved within each split, and seeds are non-overlapping so the three splits share no samples. The network-side hyperparameters listed above ($\lambda_{1}$, $\lambda_{2}$, dropout rate, and the early stopping schedule) are selected exclusively on the synthetic validation split. The post-processing parameters ($\sigma_{\mathrm{base}},\sigma_{r},\gamma$ and the outlier-correction window/threshold) are fixed implementation settings and are kept unchanged for all final evaluations; no real-world data are used to tune them. The synthetic test split is held out for the synthetic-curve validation reported in Section 3.2.4. The HDR free-form surface dataset and the silicon-wafer dataset are used *only* at final evaluation time and do not enter model retraining or network hyperparameter selection; in particular, the silicon-wafer real data are not used to tune the smoothing or outlier-correction parameters. The synthetic curves are produced by parametric distortion models and share no acquisition or pixels with either real-world dataset, so the three sources are fully independent.

### 4.2. Simulated Free-Form Surface Dataset Experiments

#### 4.2.1. Ablation Study and Overall Performance

To validate the contribution of each module, ablation experiments are designed comparing traditional methods (Argmax, Gaussian interpolation) with each component of the proposed method. Table 2 presents the quantitative results.

The expanded ablation isolates each network-side and post-processing-side module independently. Removing CFA, SPL, the validity head, or the smoothness regularizer degrades RMSE by 18.7%, 11.3%, 7.3%, and 4.0%, respectively; the ordering is consistent with the design role of each module—the more upstream and the more distortion-specific a module is, the larger its single-module contribution. Even the worst single-module-ablated variant (RMSE = 0.018) still clearly outperforms the strongest traditional baseline (Gaussian + Bilateral, RMSE = 0.041), so each module is load-bearing rather than decorative. On the post-processing side, neither outlier correction alone (0.012) nor bilateral filtering alone (0.012) reaches the complete confidence-guided smoothing (0.010); the residual gain comes from the validity score gating the two operators together, which is the intended “first regress, then confidence-guided smoothing” coupling.

The results in Table 2 can be interpreted in terms of four aspects: (1) DAFDR-Net without post-processing already reduces RMSE to 0.015; distortion-augmented training and physics-guided network design jointly enable the network to localize true peak positions from distorted curves. (2) Comparing DAFDR-Net w/o CFA (RMSE = 0.018) with the complete DAFDR-Net (RMSE = 0.015), removing the Channel-wise Feature Attention (CFA) module increases RMSE by approximately 18.7% relative to the full network, validating the usefulness of reweighting temporally encoded feature channels under distorted focus-curve patterns. (3) Bilateral filtering [31] further smooths isolated noise points by leveraging spatial neighborhood information. (4) Confidence-guided smoothing enhances smoothing in low-confidence regions, ultimately reducing RMSE to 0.010. Notably, traditional Gaussian interpolation with bilateral filtering (RMSE = 0.041) still underperforms compared to our method, indicating that the performance improvement primarily originates from the physics-guided depth regression network’s robust handling of distorted curves rather than post-processing smoothing alone.

**Figure 4:**
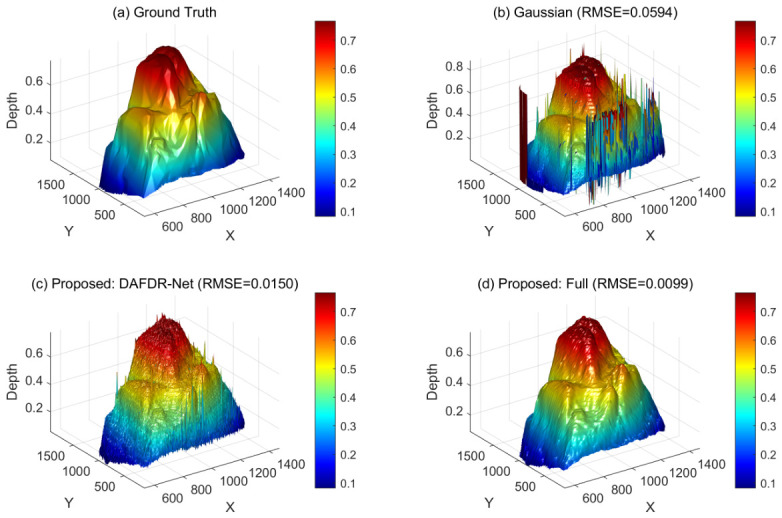
3D depth map reconstruction comparison. (**a**) Ground truth; (**b**) Gaussian interpolation (RMSE = 0.059); (**c**) Our DAFDR-Net (RMSE = 0.015); (**d**) Our complete pipeline (RMSE = 0.010).

Figure 4 presents the 3D depth map reconstruction results for different methods. Gaussian interpolation exhibits significant depth anomalies at edge regions (Figure 4b), which are effectively suppressed by our method (Figure 4c,d).

**Figure 5:**
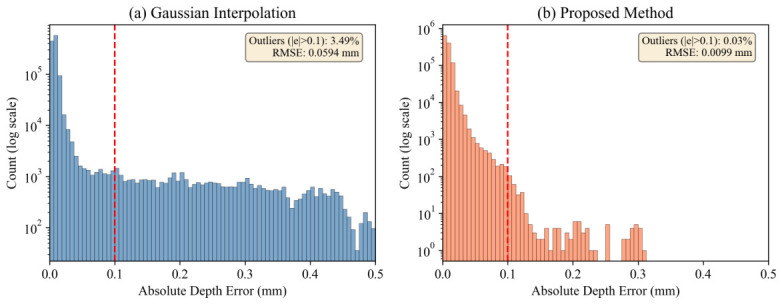
Depth absolute error distribution histogram comparison (logarithmic vertical axis). The red dashed line marks the outlier threshold |e|=0.1 mm. (**a**) Gaussian interpolation; (**b**) our method.

Extended error-distribution statistics are moved to the Supplementary Materials. The key result is that the complete pipeline reduces the 99th-percentile absolute error from 0.181 for Gaussian interpolation to 0.033 and lowers the maximum absolute error from 0.791 to 0.126, confirming that the improvement is driven by suppression of the heavy-tail outliers visible in Figure 5.

Figure 5 shows the depth error distribution histograms (logarithmic vertical axis). Gaussian interpolation still has approximately 3.5% of pixel counts to the right of the outlier threshold (|e|>0.1), exhibiting a significant long tail. Our method reduces the outlier ratio to 0.03% (approximately 100× compression), with errors highly concentrated in the small-value region.

#### 4.2.2. Robustness Analysis

To further analyze the processing effectiveness of DAFDR-Net on different distortion types, statistical analysis is conducted from two perspectives: FM curve characteristics and spatial position.

**FM Curve Type Analysis.** Table 3 presents pixel classification statistics based on FM curve characteristics.

DAFDR-Net outperforms Gaussian interpolation across all curve types. Although hard samples (asymmetric distortion, ghost peaks) constitute only approximately 11%, their Gaussian interpolation RMSE is 7–9 times that of normal samples, constituting the primary source of overall error. Our method achieves 78–83% error compression on these hard samples, clearly demonstrating the effectiveness of the distortion-aware training strategy in improving the network’s capability to handle challenging curves.

**Edge Region Analysis.** When the FM window crosses depth discontinuity boundaries, outlier errors are likely to occur. A supplementary edge/interior split shows that Gaussian interpolation has higher RMSE in edge regions (0.104) than in interior regions (0.054), with an edge outlier rate of 6.5%. Our method reduces edge-region RMSE to 0.017 and the outlier rate to 0.3%, significantly improving depth estimation stability near discontinuities.

**Figure 6:**
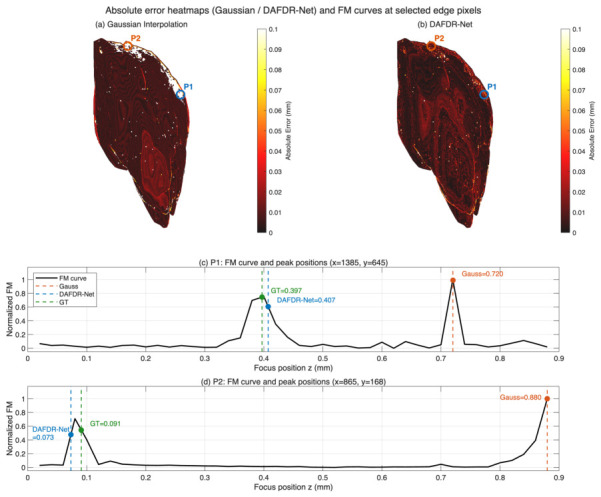
Edge region error heatmap and FM curve analysis. (**a**,**b**) Depth absolute error heatmaps for Gaussian interpolation and DAFDR-Net, with circles marking selected pixels P1 and P2; (**c**,**d**) FM curves for P1 and P2 with peak position annotations for each method (Gauss: Gaussian interpolation; DAFDR-Net: our method; GT: ground truth). All depth values shown in panels (**c**,**d**) are in millimeters and refer to the actual analyzed pixels P1 and P2; numerical comparisons in the text are based on the same values.

Figure 6 presents the error heatmaps and FM curve analysis for two representative edge pixels.

Figure 6c,d reveal two typical failure modes of Gaussian interpolation in edge regions. The FM curve of P1 exhibits a sharp anomalous peak on the right side of the sequence (single-frame anomalous response caused by FM window crossing depth boundaries); Gaussian interpolation locks onto this spurious peak (Gauss = 0.720 mm), deviating from the ground truth (GT = 0.397 mm) by more than 0.32 mm. The FM curve of P2 shows a monotonic increase toward the sequence end without a complete peak (boundary truncation effect); Gaussian interpolation is forced to select the boundary extreme value (Gauss = 0.880 mm), severely deviating from the ground truth (GT = 0.091 mm). Both distortion types share the common characteristic of FM windows crossing depth discontinuity boundaries, consistent with the “ghost peak” distortion pattern described in Section 3.2.1.

Our method successfully localizes depths close to the ground truth at both points (P1: 0.407 mm, error 0.010 mm; P2: 0.073 mm, error 0.018 mm). This result validates the effectiveness of the physics-guided distortion-aware training strategy in Section 3.2: P1’s failure mode (anomalous spike in edge region) has a similar curve morphology to the “ghost peak” type in training data. The network, exposed to various distortion samples during training, learns the ability to localize true peaks from overall curve morphology; the CFA module reweights feature channels learned from such distorted temporal responses, while the SPL mechanism provides geometric inductive bias.

#### 4.2.3. Comparison with Existing Methods

The MRF depth map optimization method proposed by He et al. [11] is a representative work for HDR surface SFF reconstruction, employing an energy function minimization framework and designing a bad-point detection mask based on fitting residuals. Table 4 compares the two methods under the same dataset and evaluation protocol.

Our method reduces RMSE from 0.016 (MRF optimized result) to 0.010 (36.5% reduction). The core difference between the two methods lies in distortion handling strategy: the MRF method detects bad points in the post-processing stage and relies on neighborhood smoothing terms for filling, with interpolation quality limited when bad points appear in clusters; our method uses DAFDR-Net to directly regress correct depths from distorted curves, reducing the number of pixels requiring post-processing remediation from the source. In terms of computational efficiency, our method has a total processing time of approximately 3.0 s (RTX 3090, NVIDIA Corporation, Santa Clara, CA, USA), approximately 3.3× faster than MRF iterative optimization (approximately 10 s). As a lightweight 1D-CNN, DAFDR-Net inference accounts for only ≈13% of total time, with computational complexity linear in sequence length *K*, demonstrating good scalability for long-sequence scenarios.

Table 5 reports a stage-level runtime breakdown of both pipelines on the same hardware (RTX 3090, 2000×2000 image, K=44). The MRF baseline is dominated by the iterative energy minimization (8.00 s, ≈81% of its total), so its 3.3× slowdown over our pipeline is concentrated in a single irreducible stage. In our pipeline, by contrast, no single stage exceeds ≈40% of the total, and DAFDR-Net inference itself takes only 0.38 s (≈13%); the network is a lightweight 1D-CNN whose per-pixel cost is linear in the focal-stack length *K*. On the silicon-wafer dataset (K=3500) the measured total processing time grows to ≈18 s, in line with this linear-*K* scaling.

### 4.3. Real-World Dataset Experiments

#### 4.3.1. Depth Reconstruction Results and Analysis

This section validates the industrial applicability of the proposed method on monocrystalline silicon wafer data. As described in Section 4.1, the primary challenge of this data stems from low signal-to-noise ratios caused by weak textures, rather than HDR saturation. Representative raw frames are provided in the Supplementary Materials.

**Figure 7:**
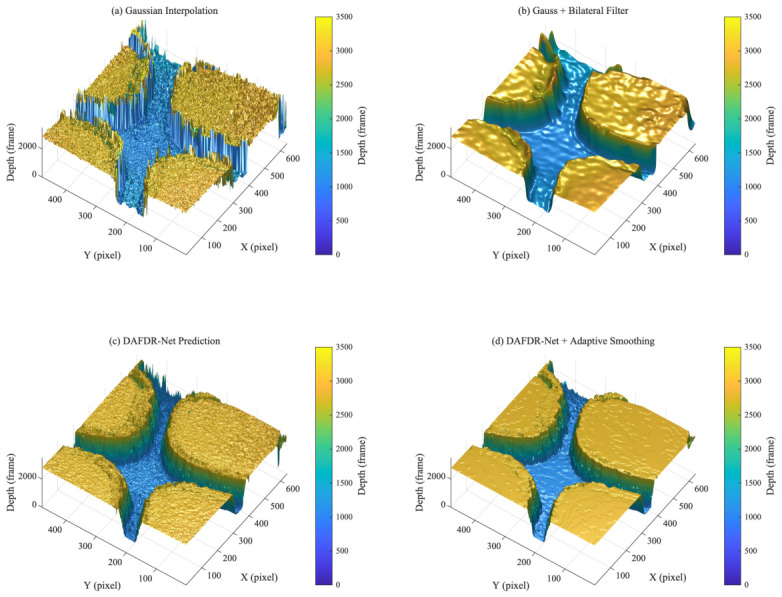
Silicon wafer depth map 3D visualization comparison. (**a**) Gaussian interpolation; (**b**) Gaussian interpolation + bilateral filter; (**c**) DAFDR-Net initial result; (**d**) DAFDR-Net + confidence-guided adaptive smoothing. The silicon wafer mesa edge exhibits extensive weak-texture regions where low focus curve SNR and peak misjudgment cause spikes and pseudo-undulations in the depth map. Our method suppresses such spikes through the “first regress, then confidence-guided adaptive smoothing” strategy while maintaining natural transition at mesa edges.

Figure 7 presents 3D depth reconstruction results comparing four methods: Gaussian interpolation (unsmoothed), Gaussian interpolation + bilateral filter (traditional post-processing), DAFDR-Net initial result, and DAFDR-Net + confidence-guided adaptive smoothing (our complete method).

The 3D visualization results show:

1. **Gaussian interpolation (Figure 7a)**—The weak-texture regions at the mesa edge produce numerous spikes and local pseudo-structures.
2. **Gaussian interpolation + bilateral filter (Figure 7b)**—Uniform-parameter smoothing suppresses spikes to some extent, but local anomalous points remain, and the overall topography is affected by non-uniform smoothing side effects.
3. **DAFDR-Net (Figure 7c)**—Distortion-aware depth regression significantly reduces misjudgments in weak-texture regions, with overall noise level decreased. This improvement benefits from the “Low-SNR weak peak” training data designed in Section 3.2.1: the network learns the ability to localize true depths when weak peaks coexist with strong noise. However, per-pixel independent prediction may still produce a few isolated spikes and local discontinuities.
4. **DAFDR-Net + confidence-guided adaptive smoothing (Figure 7d)**—Confidence-driven differential smoothing enhances smoothing in low-reliability regions while suppressing over-smoothing in high-reliability regions. Spikes and outliers are further eliminated, with mesa and groove regions becoming more uniform and consistent.

**Figure 8:**
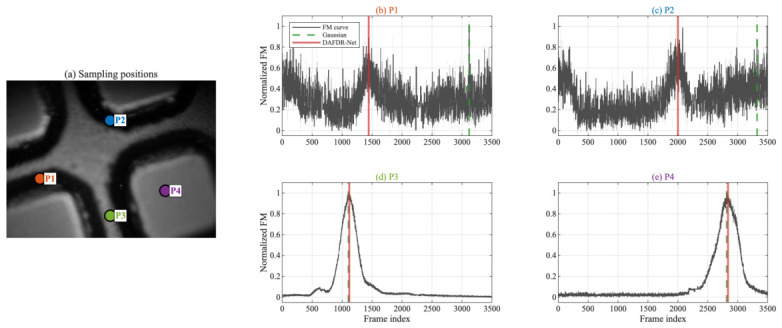
FM curve distortion analysis in weak-texture regions of the silicon wafer. (**a**) Sampling point locations; (**b**–**e**) Normalized FM curves and depth estimation results for P1–P4. Green dashed lines indicate Gaussian interpolation peak positions; red solid lines show DAFDR-Net estimated depths.

To further reveal the source of the above depth reconstruction differences, four representative pixels are selected for FM curve analysis (Figure 8). P1 and P2 are located in the mesa edge transition region, where texture is extremely weak and illumination is insufficient due to shadow occlusion at the interface between the mesa protrusion and groove. P3 and P4 are located in the groove interior and mesa center, respectively, with relatively clear textures.

Figure 8b,c reveal typical distortion patterns of FM curves in weak-texture regions. The FM curves of P1 and P2 exhibit high-frequency noise-dominated characteristics without obvious single peaks: normalized FM values fluctuate dramatically between 0.1–0.8, with true focus peaks submerged in the noise floor. This distortion pattern closely matches the “Low-SNR weak peak” type described in Section 3.2.1—weak focus response (small α) combined with significant additive noise (large $\sigma_{n}$), resulting in extremely low signal-to-noise ratios. Gaussian interpolation fails on such curves: P1’s Gaussian interpolation result (frame 3120) locks onto a noise-spurious peak in the latter part of the sequence; P2’s Gaussian interpolation result (frame 3323) is offset to the sequence end, both severely deviating from true depth positions. In contrast, DAFDR-Net outputs frame 1443 and frame 2001 for P1 and P2, respectively, successfully localizing to reasonable depth intervals. This result validates the effectiveness of the distortion-aware training strategy: the network, exposed to large quantities of “Low-SNR weak peak” synthetic samples during training, learns the ability to identify weak true peaks from noise-dominated curves.

Figure 8d,e show FM curve characteristics in regions with better texture. The FM curves of P3 (groove region) and P4 (mesa region) both exhibit standard unimodal shapes with clear peaks and stable baselines, consistent with the ideal Gaussian distribution assumption. On such normal samples, Gaussian interpolation and DAFDR-Net depth estimation results are highly consistent (P3: both approximately frame 1120; P4: both approximately frame 2837), indicating that DAFDR-Net does not sacrifice depth estimation accuracy on normal samples while handling hard samples.

The above analysis indicates that the primary challenge of silicon wafer data is concentrated in the mesa edge transition regions: in these areas, extremely low FM curve signal-to-noise ratios due to weak textures and insufficient illumination cause traditional Gaussian interpolation to frequently lock onto noise spurious peaks, resulting in spikes and outliers in depth maps (corresponding to the edge region anomalous depths in Figure 7a). DAFDR-Net acquires the ability to localize true depths from low-SNR curves through physics-guided network design and distortion-aware training: the CFA module reweights noise-sensitive temporal-response feature channels, and the SPL mechanism guides the network to focus on potential true peak positions, reducing the number of pixels requiring post-processing remediation from the source. This is the key to our method’s performance improvement in weak-texture scenarios.

#### 4.3.2. Quantitative Evaluation

Given the topographic characteristics of the silicon wafer’s two-layer structure, internal consistency metrics are employed to evaluate reconstruction quality, including flat region standard deviation (measuring noise level) and two-layer separation (measuring depth estimation consistency). Table 6 presents quantitative comparison results.

Table 6 shows that DAFDR-Net already significantly reduces noise levels compared to traditional methods, and confidence-guided adaptive smoothing further improves internal consistency: flat region standard deviation decreases from 88.0 frames to 42.9 frames (51.3% reduction), with depth estimation for both mesa and groove layers becoming more stable.

**Figure 9:**
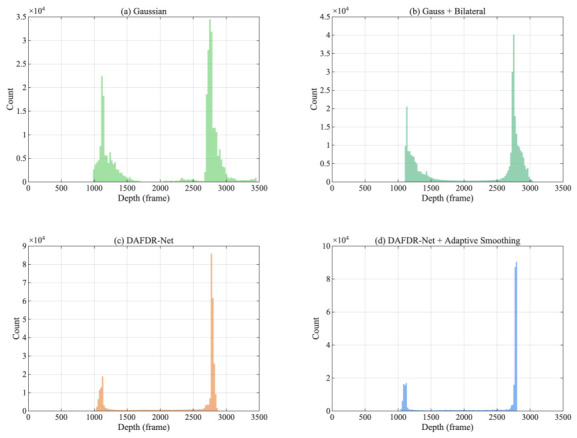
Depth distribution histogram comparison. (**a**) Gaussian interpolation; (**b**) Gaussian interpolation + bilateral filter; (**c**) DAFDR-Net; (**d**) DAFDR-Net + adaptive smoothing. From (**a**–**d**), the bimodal peaks progressively narrow and inter-peak pixels decrease, showing depth estimation transitioning from “multiple spikes/misjudgments” to a more consistent two-layer structure.

Extended ISO 25178 areal roughness statistics are reported in the Supplementary Materials. In brief, the complete pipeline reduces $S_{a}$ by approximately 60% on the mesa plateau and 47% on the groove plateau relative to the Gaussian + BF baseline; the residual distribution also becomes less asymmetric and less heavy-tailed, consistent with the histogram comparison in Figure 9.

Figure 9 presents depth distribution histogram comparisons for the four methods, intuitively reflecting differences in bimodal separation and outlier tail distribution.

Depth profile comparisons along two cross-sections are provided in the Supplementary Materials; they show the same trend as Figure 9: confidence-guided adaptive smoothing suppresses spikes in low-confidence regions while preserving natural transitions at mesa edges.

#### 4.3.3. Quantitative Step-Height Error Against an External Reference

To address the absence of a quantitative ground truth on the silicon-wafer dataset, the mesa–groove step was independently profiled with a Zeiss LSM 900 laser-scanning confocal microscope (Carl Zeiss AG, Oberkochen, Germany), giving a reference step height $h_{\mathrm{ref}}=170.3\;\mu$m. Because the LSM 900 and the SFF system differ in lateral resolution, field of view, and sampling density, we restrict the external-reference comparison to quantities for which co-registration is reliable: a representative cross-section through the mesa–groove transition and a 15-pixel-wide band along the detected mesa–groove boundary. We then compute four external-reference metrics for each pipeline, in the same order as the columns of Table 7: (i) step-height error, defined as $|\hat{h}-h_{\mathrm{ref}}|$, where $\hat{h}$ is the mesa-minus-groove plateau-median difference estimated by each SFF pipeline; (ii) profile RMSE along the representative cross-section (in μm, with Δz=0.1μm/frame); (iii) edge RMSE within the 15-pixel band around the mesa–groove boundary detected on the registered LSM 900 reference profile; and (iv) outlier rate, defined as the fraction of pixels whose depth deviates by more than 3σ from the local plateau median in a 21×21 window.

The complete pipeline reduces the step-height error from 7.5μm for Gaussian interpolation to 0.3μm, the profile RMSE from 14.2μm to 2.67μm, the edge RMSE from 19.9μm to 4.87μm, and the outlier rate from 12.8% to 0.4%. The two RMSE columns and the per-plateau standard deviations in Table 6 (reported in frames, equivalent to μm at Δz=0.1μm/frame) capture different quantities—the profile RMSE is taken along a single mesa–groove cross-section against the LSM 900 reference, the edge RMSE is restricted to a 15-pixel band around the boundary, and the plateau std is computed over the entire flat regions—so they are not expected to match numerically; the ranking across methods, however, is consistent across all three. The residual step-height error for DAFDR-Net + AS is much smaller than the per-pixel plateau noise because plateau medians average over ∼$10^{4}$ pixels. Together with the qualitative comparison in Section 4.3.1 and the histogram analysis in Figure 9, these results indicate that the qualitative gain on the silicon-wafer data corresponds to a real metrological improvement at the ∼170 μm step regime.

### 4.4. Limitations and Generalization

The two datasets evaluated in this work cover qualitatively different distortion regimes—HDR saturation and ghost peaks on the free-form surface, weak texture and low SNR on the silicon wafer—and the consistent improvement on both is the principal cross-scenario evidence we offer for the proposed physics-guided design. Nonetheless, the network has only been validated within a finite range of optical configurations, and several limitations should be made explicit.

Changes in optical configuration (aperture, magnification, depth of field) modify the ratio $\sigma_{\mathrm{DoF}}/\Delta z$ that Equation (4) keys off of. Adaptation in this case proceeds by recomputing the convolution kernel sizes via Equation (4) and retraining on parametrically regenerated synthetic data of matching sequence length; no real-world annotations are required. Changes in sensor type (saturation behavior, lateral blooming extent, noise color) shift the empirical distribution of distortion patterns. The synthesis class proportions specified in Section 3.2.1 can be re-tuned for a different sensor, while the CFA module and validity head should be treated as reusable architectural components rather than sensor-invariant calibrated predictors.

Architectural limits of the present design that are not removed by retraining alone include:

- **Single-peak SPL.** The soft peak localization mechanism (Equation (6)) assumes a single dominant peak; bimodal distributions arising from transparent or semi-transparent multi-surface targets fall outside the present scope. Extending to such targets requires replacing the single-depth regression head with a multi-peak detection head and adding a “true bimodal” synthetic class [36].
- **Per-configuration fixed kernels.** Kernel sizes are determined per acquisition configuration via Equation (4) and do not adapt within a single trained model; dilated [39] and deformable/dynamic kernels [40] are a future direction for spanning multiple DoF regimes with one model.
- **Synthetic-only training.** The synthetic distortion model captures saturation, ghost peaks, low-SNR weak peaks, asymmetry, and background noise but does not yet model lateral electron-overflow blooming, fixed-pattern noise, or sensor banding; CFA provides only indirect feature-level mitigation against these effects rather than explicit frame-wise or spatial blooming correction.
- **Sub-quantization-noise textures.** On surfaces whose texture variation lies below the sensor quantization-noise floor, the focus curve carries no defocus signal in principle; this is an information-theoretic bound, not a network failure. The validity head ${\hat{m}}_{p}$ flags such pixels rather than fabricating a depth, and the confidence-guided smoothing replaces them by neighborhood interpolation (Section 3.3).

## 5. Conclusions

This paper presented a distortion-aware SFF reconstruction pipeline for opaque single-surface targets under HDR and weak-texture conditions. The central contribution is to move robustness upstream: instead of only smoothing an already erroneous depth map, DAFDR-Net learns physically motivated distorted focus curves and estimates both depth and validity. The CFA module reweights temporally encoded feature channels, SPL keeps the regression tied to a peak-localization prior, and confidence-guided smoothing uses the validity output to repair low-reliability pixels without uniformly blurring reliable regions.

The experiments support three specific conclusions. First, on the HDR free-form surface dataset, the complete pipeline achieves RMSE = 0.010, reducing error by 36.5% relative to the reported MRF-optimized result and by 83.4% relative to Gaussian interpolation. Second, the improvement is not only an average-error effect: the 99th-percentile absolute error decreases from 0.181 to 0.033, and representative edge pixels show that the network avoids spurious-peak locking. Third, on weak-textured silicon wafer data, the flat-region standard deviation decreases from 88.0 frames for Gaussian + BF to 42.9 frames, indicating more stable two-layer reconstruction.

The method should therefore be interpreted as a practical and lightweight solution for the evaluated HDR and weak-texture SFF configurations, not as a universal depth solver. Its current assumptions are a single dominant focus peak, an opaque single-surface target, and retraining when the optical depth-of-field-to-step ratio or sensor distortion statistics change substantially. Future work will focus on DoF-adaptive kernels, explicit spatial blooming and sensor-noise models, multi-peak depth prediction for transparent or semi-transparent layers, and domain adaptation using limited real annotated samples.

## Figures and Tables

**Table 1:** Comparison of representative SFF method families. “Distortion handling” refers to the explicit treatment of saturation, ghost peaks, or low-SNR weak peaks; “Confidence” refers to a per-pixel reliability score that downstream stages can use.

Method Family	Core Model	Distortion Handling	Confidence	Spatial Prior	Training Data
Argmax/Gaussian Interp. [8,18]	Analytic peak localization	Symmetric unimodal assumption	None	None	N/A
Local filtering (BF/Guided) [31,32]	Post-processing filter	None upstream	None	Local linear	N/A
MRF post-processing [11,23]	Graphical energy minimization	Bad-point flag from fit residual	Implicit (residual)	Global energy min.	N/A
FocusNet-style FC [19]	Fully connected regressor	Ideal Gaussian only	None	None	Ideal curves
**DAFDR-Net (Ours)**	**Physics-guided 1D-CNN + CFA/SPL**	**5 explicit synthetic distortion classes**	**Validity head + curve sharpness**	**Confidence-guided smoothing**	**Synthetic distorted curves**

Bold font highlights the proposed method (DAFDR-Net).

**Table 2:** Ablation Study Results (RMSE and MAE in mm; computed on the valid ground-truth mask of the simulated free-form surface dataset, scanning range 0.02–0.88 mm).

Method	RMSE	MAE	vs. Gaussian
Argmax	0.060	0.019	+0.5%
Gaussian Interpolation	0.059	0.018	Baseline
Gaussian + Bilateral Filter	0.041	0.014	−30.5%
DAFDR-Net w/o CFA	0.018	0.010	−70.0%
DAFDR-Net w/o SPL	0.017	0.009	−71.9%
DAFDR-Net w/o Validity Head	0.016	0.009	−72.9%
DAFDR-Net w/o $L_{\mathrm{smooth}}$	0.016	0.009	−73.7%
DAFDR-Net (Ours)	0.015	0.009	−74.7%
DAFDR-Net + Outlier Corr. only	0.012	0.008	−79.1%
DAFDR-Net + Bilateral	0.012	0.007	−80.5%
**DAFDR-Net + Conf. Smooth**	** 0.010 **	** 0.007 **	**−83.4%**

Bold font indicates the best result in each column.

**Table 3:** Error Statistics for Different FM Curve Types (RMSE in mm).

Curve Type	Pixel %	Gaussian RMSE	DAFDR-Net RMSE	Improvement
Normal Single-peak	88.9%	0.021	0.011	−47.1%
Asymmetric Distortion	3.9%	0.192	0.032	−83.2%
Ghost/Multi-peak	7.2%	0.152	0.033	−78.2%

**Table 4:** Comparison with MRF Method (RMSE and MAE in mm).

Method	RMSE	MAE	Rel. Improvement
He et al. [11] (Unoptimized)	0.038	—	—
He et al. [11] (MRF Optimized)	0.016	—	−58.8%
**Ours**	** 0.010 **	** 0.007 **	**−73.9%**

Bold font indicates the best result in each column.

**Table 5:** Stage-level runtime breakdown on the HDR free-form surface dataset (RTX 3090, 2000×2000 image, K=44).

Stage	Ours (s)	MRF [11] (s)
Wavelet FM extraction	1.20	1.20
Initial peak (Argmax/Gaussian)	0.05	0.10
DAFDR-Net inference	0.38	—
Confidence map computation	0.18	—
Adaptive bilateral filtering	0.72	—
Outlier correction/bad-point handling	0.30	0.50
MRF energy minimization (50 iter)	—	8.00
I/O & miscellaneous	0.17	0.05
**Total**	**3.00**	**9.85**

Bold font marks the total processing time of each pipeline.

**Table 6:** Silicon Wafer Depth Map Quantitative Metrics Comparison (values are depth standard deviations in units of frame index *k*, with K=3500 frames over the scan range).

Metric	Gaussian + BF	DAFDR-Net	DAFDR-Net + AS	Improvement
Flat Region Std. Dev.	88.0	53.1	** 42.9 **	**−51.3%**
Groove Layer Std. Dev.	62.4	38.6	** 28.8 **	**−53.8%**
Mesa Layer Std. Dev.	73.3	36.0	** 26.7 **	**−63.6%**

Bold font indicates the best result for each metric.

**Table 7:** Silicon-wafer quantitative metrics referenced to a Zeiss LSM 900 laser-scanning confocal microscope ($h_{\mathrm{ref}}=170.3\;\mu$m). The “Measured $\hat{h}$” column is the mesa-minus-groove plateau-median difference estimated by each SFF pipeline, compared against the external reference. “AS” denotes confidence-guided adaptive smoothing (the same configuration as the “Conf. Smooth” row in Table 2).

Method	Measured $\hat{h}$ (μm)	Step-Height Err. (μm)	Profile RMSE (μm)	Edge RMSE (μm)	Outlier Rate (%)
Gaussian Interpolation	162.8	7.5	14.2	19.9	12.8
Gaussian + Bilateral Filter	166.0	4.3	8.79	13.4	7.4
DAFDR-Net	169.6	0.7	3.60	7.18	1.9
**DAFDR-Net + AS**	**170.0**	**0.3**	**2.67**	**4.87**	**0.4**

Bold font indicates the best result in each column.

## Data Availability

The source code for the proposed DAFDR-Net, including the core network architecture with Channel-wise Feature Attention (CFA) and Soft Peak Localization (SPL) modules, loss functions, focus measure operators, and post-processing pipeline, is publicly available at https://github.com/awsl666wuhu/DAFDR-Net (accessed on 14 April 2026).

## Supplementary Materials

The following supporting information can be downloaded at https://www.mdpi.com/article/10.3390/s26113390/s1, Figure S1: Overall pipeline of the proposed method; Figure S2: Variable-focus surface topography measurement system; Figure S3: Example frames from the silicon wafer multi-focus image sequence; Figure S4: Depth profile comparison along two representative silicon-wafer profiles; Table S1: DAFDR-Net architecture parameter summary; Table S2: Kernel-size sweep on the HDR free-form surface dataset; Table S3: Network-depth sweep on the HDR free-form surface dataset; Table S4: Training-data proportion sensitivity on the HDR free-form surface dataset; Table S5: Depth estimation performance comparison on synthetic focus-curve data; Table S6: Extended error-distribution statistics for the free-form HDR dataset; Table S7: Error comparison between edge and interior regions; Table S8: Extended areal-roughness and error-shape statistics on silicon-wafer plateaus.

## Abbreviations

The following abbreviations are used in this manuscript:

SFF	Shape-from-Focus
HDR	High Dynamic Range
FMO	Focus Measure Operator
CFA	Channel-wise Feature Attention
SPL	Soft Peak Localization
DAFDR-Net	Distortion-Aware Focal Depth Regression Network
MRF	Markov Random Field
RMSE	Root Mean Square Error
MAE	Mean Absolute Error
SNR	Signal-to-Noise Ratio
BN	Batch Normalization
GAP	Global Average Pooling
BCE	Binary Cross-Entropy
MSE	Mean Squared Error
