# Supplementary material for: Robust Shape-from-Focus via Physics-Inspired Distortion-Aware Focal Depth Regression
Source: Sensors (Basel). 2026 May 27;26(11):3390. doi: 10.3390/s26113390 (PMC13258936; doi:10.3390/s26113390)
Supplement: Supplementary file 1 [file sensors-26-03390-s001.zip › sensors-4288310-supplementary.pdf]

sensors

MDPI

Article

Robust Shape-from-Focus via Physics-Inspired Distortion-Aware Focal Depth Regression

Xin Li [1], Wei Shen [1], Jian Li [2], Zhongsheng Zhai [1], Xuhong Guan [1] and Zili Lei [1,*]

1 Hubei Key Laboratory of Modern Manufacturing Quantity Engineering, School of Mechanical Engineering, Hubei University of Technology, Wuhan 430068, China; 18171870097@163.com (X.L.); 102400017@hbut.edu.cn (W.S.); zs.zhai@hbut.edu.cn (Z.Z.); 15327958028@163.com (X.G.)

2 School of Mechanical and Electrical Engineering, Changsha University, Changsha 410022, China; z20190620@ccsu.edu.cn

* Correspondence: 20231136@hbut.edu.cn

S1. Baseline and Evaluation Equations

For completeness, this section lists the conventional baseline and metric equations that were moved from the main manuscript to reduce redundancy.

Discrete maximum search estimates the best-focus frame as

$$\hat{d}_p^{\text{argmax}} = \arg\max_{1 \le k \le K} f_p(k). \tag{S1}$$

Gaussian interpolation improves sub-frame localization by fitting the three-point neighborhood of the discrete maximum [1–3]. Let the discrete peak be $k_m = \hat{d}_p^{\text{argmax}}$, and let (f_1, f_2, f_3) be the focus-measure values at $(k_m - 1, k_m, k_m + 1)$. The interpolated peak is

$$\hat{d}_p^{\text{Gauss}} = k_m + \frac{\ln f_1 - \ln f_3}{2(\ln f_1 - 2\ln f_2 + \ln f_3)}. \tag{S2}$$

A representative MRF-style post-processing objective can be written as

$$E(I) = \sum_p \left(S'(p) - I(p)\right)^2 + \lambda \sum_{(p,q)\in\mathcal{N}} \omega_{(p,q)} \left(I(p) - I(q)\right)^2, \tag{S3}$$

where S' and I denote the observed and optimized depth maps, respectively, $\mathcal{N}$ is the neighborhood set, and λ is the regularization coefficient.

The simulated free-form surface dataset uses Root Mean Square Error (RMSE) and Mean Absolute Error (MAE):

$$\text{RMSE} = \sqrt{\frac{1}{|\mathcal{M}|} \sum_{p\in\mathcal{M}} \left(D(p) - D^*(p)\right)^2}, \tag{S4}$$

$$\text{MAE} = \frac{1}{|\mathcal{M}|} \sum_{p\in\mathcal{M}} |D(p) - D^*(p)|, \tag{S5}$$

where $D(p)$ is the estimated depth, $D^*(p)$ is the ground truth depth, and $\mathcal{M}$ is the valid evaluation mask.

For the extended simulated-data statistics, the absolute error is defined as $|e(p)| = |D(p) - D^*(p)|$ on $\mathcal{M}$. $P_{95}|e|$ and $P_{99}|e|$ denote the 95th and 99th percentiles of $|e(p)|$, respectively; Max $|e|$ is the largest absolute error; and Error Std. is the standard deviation of $e(p)$ over $\mathcal{M}$. For the silicon-wafer plateaus, the residual within a mesa or groove region $\mathcal{R}$ is computed after subtracting the regional mean depth. The ISO 25178 roughness indicators

Check for updates

Academic Editor: Michele Norgia

Received: 14 April 2026
Revised: 15 May 2026
Accepted: 21 May 2026
Published: 27 May 2026

Copyright: © 2026 by the authors. Licensee MDPI, Basel, Switzerland. This article is an open access article distributed under the terms and conditions of the Creative Commons Attribution (CC BY) license.

are computed as $S_a = |\mathcal{R}|^{-1} \sum_{p \in \mathcal{R}} |D(p) - \bar{D}_{\mathcal{R}}|$ and $S_q = \sqrt{|\mathcal{R}|^{-1} \sum_{p \in \mathcal{R}} (D(p) - \bar{D}_{\mathcal{R}})^2}$; skewness and kurtosis are computed from the same residual distribution.

S2. Network, Loss, and Post-Processing Details

Table S1. DAFDR-Net architecture parameter summary.

Module	Layer Configuration	Output Dim	#Params	Physical Meaning/Design Rationale
Input	Normalized FM sequence $\tilde{f}_p(k)$	$1 \times K$	—	Defocus response curve
Defocus Encoding I	Conv1D(1→16, k=5) + BN + ReLU	$16 \times K$	128	Coarse-scale peak shape features
Defocus Encoding II	Conv1D(16→32, k=3) + BN + ReLU	$32 \times K$	1,632	Fine-scale local features
CFA-Squeeze	GAP_K + FC(32→16) + ReLU	16	528	Temporal pooling and channel compression
CFA-Excite	FC(16→32) + Sigmoid	32	544	Channel-wise feature-attention weights $\mathbf{r} \in \mathbb{R}^{32}$
CFA-Scale	$F'_{c,k} = r_c F_{c,k}$	$32 \times K$	0	Reweighting of temporal-response feature channels
SPL + Aggregation	Softmax + Flatten + FC(32K→128) + ReLU + Dropout	128	$32K \times 128 + 128$	Soft peak localization
Depth Head	FC(128→64→1) + Sigmoid	1	8,321	Normalized depth $\hat{d}_p \in [0, 1]$
Validity Head	FC(128→64→1)	1	8,321	Foreground mask probability $\hat{m}_p$

Note: Parameter count computed with $K = 44$, yielding approximately 200k total parameters. CFA is based on the SE mechanism [4] and outputs channel-wise weights after temporal pooling, rather than explicit per-frame reliability weights; this notation is consistent with the CFA equation in the main manuscript. SPL provides geometric inductive bias through differentiable soft peak localization.

The loss terms in the main manuscript are defined as

$$\mathcal{L}_{\text{BCE}} = \text{BCE}(m, \hat{m}), \quad \mathcal{L}_{\text{MSE}} = \text{MSE}(\hat{d}, d \mid m = 1), \tag{S6}$$

$$\mathcal{L}_{\text{smooth}} = \frac{1}{K-1} \sum_{k=1}^{K-1} |h_{k+1} - h_k|. \tag{S7}$$

The confidence-guided adaptive smoothing module applies a bilateral-filter form

$$D_{\text{smooth}}(p) = \frac{\sum_{q \in \mathcal{N}(p)} w_s(p,q) \cdot w_r(p,q) \cdot D(q)}{\sum_{q \in \mathcal{N}(p)} w_s(p,q) \cdot w_r(p,q)}, \tag{S8}$$

where $\mathcal{N}(p)$ is the local neighborhood, $w_s(p,q)$ is the spatial weight, and $w_r(p,q)$ is the depth similarity weight. The spatial scale is confidence adaptive:

$$\sigma_s(p) = \sigma_{\text{base}} \cdot (1 + \gamma \cdot (1 - C(p))). \tag{S9}$$

To suppress isolated outliers, the depth gradient magnitude is first computed as

$$G(p) = \sqrt{\left(\frac{\partial D}{\partial x}\right)^2 + \left(\frac{\partial D}{\partial y}\right)^2}. \tag{S10}$$

Outliers are detected using the three-sigma rule [5]:

$$\mathcal{O} = \{p \mid G(p) > \mu_G + k \cdot \sigma_G\}. \tag{S11}$$

Flagged pixels are corrected by a confidence-weighted average of non-outlier neighbors:

$$D_{\text{fix}}(p) = \frac{\sum_{q \in \mathcal{N}(p) \setminus \mathcal{O}} C(q) \cdot D(q)}{\sum_{q \in \mathcal{N}(p) \setminus \mathcal{O}} C(q)}. \tag{S12}$$

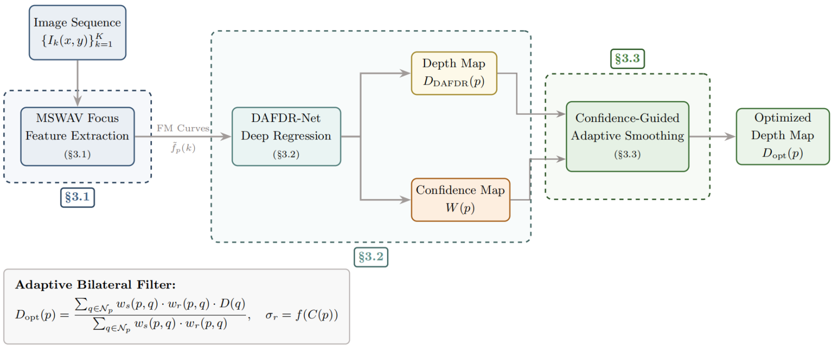

Figure S1. Overall pipeline of the proposed method. The image sequence undergoes wavelet feature extraction, DAFDR-Net outputs depth and foreground probability, and confidence-guided adaptive smoothing produces the optimized depth map.

S3. Kernel-size and Network-depth Sensitivity

To support the physics-keyed kernel-size choice in the main manuscript, we sweep the convolution-kernel pair (k_1, k_2) at fixed depth and the network depth at fixed kernels on the HDR free-form surface dataset. All other hyper-parameters are held at their main-text values.

Table S2. Kernel-size sweep on the HDR free-form surface dataset (depth = 2).

(k_1, k_2)	RMSE (mm)	MAE (mm)	Comment
(3, 3)	0.019	0.010	Under-covers response
**(5, 3) (ours)**	**0.015**	**0.009**	Matches scale rule
(7, 3)	0.016	0.009	Mild over-smoothing
(7, 5)	0.017	0.009	No gain
(9, 5)	0.020	0.011	Over-smoothing dominates

Table S3. Network-depth sweep on the HDR free-form surface dataset (kernels fixed at $(k_1, k_2) = (5, 3)$).

Depth	Params	RMSE (mm)	MAE (mm)
1 layer	116 k	0.023	0.012
**2 layers (ours)**	**200 k**	**0.015**	**0.009**
3 layers	282 k	0.015	0.009
4 layers	364 k	0.016	0.009

Note: parameters are reported for $K = 44$ and grow approximately linearly with sequence length. Beyond two layers, additional depth does not improve accuracy and slightly increases error, indicating that the physics-prior two-layer encoder already captures the relevant defocus geometry; the extra capacity is therefore dropped to keep the network lightweight.

S4. Training-data Proportion Sensitivity

The class proportions used in the main text (Sat 20 % / Ghost 25 % / Low-SNR 35 % / Asym 10 % / Bg 5 % / Ideal 5 %) were selected based on the observed prevalence of each distortion in HDR and weak-texture data. To verify that the strategy is not over-fitted to a specific test distribution, alternative proportions were tested by independently retraining DAFDR-Net on each variant and evaluating RMSE on the HDR free-form surface dataset.

https://doi.org/10.3390/s26113390

Table S4. Training-data proportion sensitivity on the HDR free-form surface dataset.

Configuration	Sat (%)	Ghost (%)	Low-SNR (%)	Asym (%)	Bg (%)	Ideal (%)	RMSE (mm)
Ours (baseline)	20	25	35	10	5	5	**0.015**
Sat↑	30	25	25	10	5	5	0.016
Ghost↑	15	35	30	10	5	5	0.015
Low-SNR↑	15	20	50	5	5	5	0.016
Uniform	17	17	17	17	17	15	0.016
Severe Sat	50	10	10	10	10	10	0.018

Note: "Sat↑" denotes a +10 percentage-point shift toward the saturation class compensated by a −10 percentage-point shift across the other hard classes (similarly for the other rows). Within ±10–15 percentage-point shifts of the four hard-sample classes the RMSE varies by $<8\%$ in relative terms, indicating that the strategy is not over-fitted to the test-set distribution.

S5. Auxiliary Experimental Materials

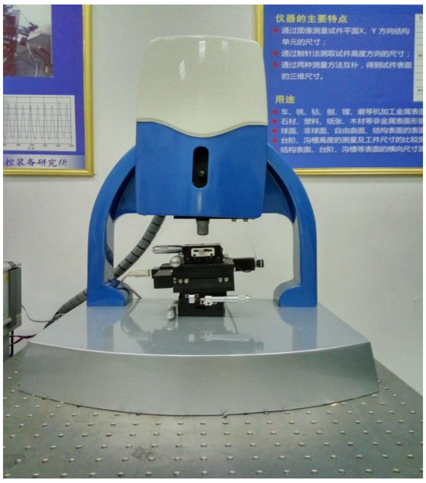

Figure S2. Variable-focus surface topography measurement system.

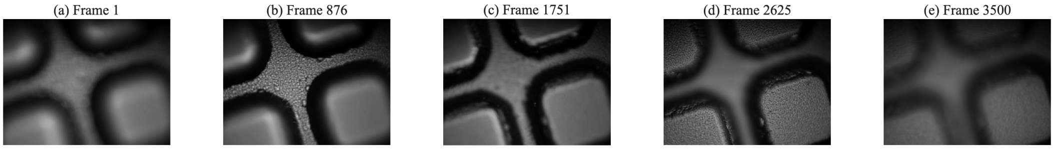

Figure S3. Example frames from the silicon wafer multi-focus image sequence.

Table S5. Depth estimation performance comparison on synthetic focus-curve data.

Method	MAE (All)	MAE (Hard)	Rel. Improvement	Mask Acc.
Argmax	0.061	0.056	—	N/A
Gaussian Interp.	0.061	0.055	Baseline	N/A
**DAFDR-Net (Ours)**	**0.021**	**0.021**	**+62.1%**	**100%**

Table S6. Extended error-distribution statistics for the free-form HDR dataset.

Method	$P_{95}\|e\|$	$P_{99}\|e\|$	Max $\|e\|$	Error Std.
Gaussian Interpolation	0.085	0.181	0.791	0.057
DAFDR-Net (Ours)	0.026	0.041	0.183	0.012
**DAFDR-Net + Conf. Smooth**	**0.022**	**0.033**	**0.126**	**0.007**

https://doi.org/10.3390/s26113390

Table S7. Error comparison between edge and interior regions.

Region	Pixel %	Gaussian RMSE	Ours RMSE	Improv.	Outlier (G)	Outlier (Ours)
Edge	7.2%	0.104	0.017	−83.3%	6.5%	0.3%
Interior	92.8%	0.054	0.009	−83.5%	3.3%	0.0%

Table S8. Extended areal-roughness and error-shape statistics on silicon-wafer plateaus (units: frames).

	**Mesa plateau**		**Groove plateau**		**Distribution shape**	
**Method**	S_a	S_q	S_a	S_q	**Skew.**	**Kurt.**
Gaussian + BF	50.4	73.3	42.5	62.4	0.94	7.6
DAFDR-Net	26.5	36.0	28.7	38.6	0.52	4.5
**DAFDR-Net + AS**	**20.6**	**26.7**	**22.4**	**28.8**	**0.31**	**3.4**

Note: S_q values match the per-region standard deviations in the main manuscript; S_a is the corresponding ISO 25178 arithmetic-mean roughness [6]. Skewness and kurtosis are computed on the depth residual within each plateau and averaged across mesa and groove regions.

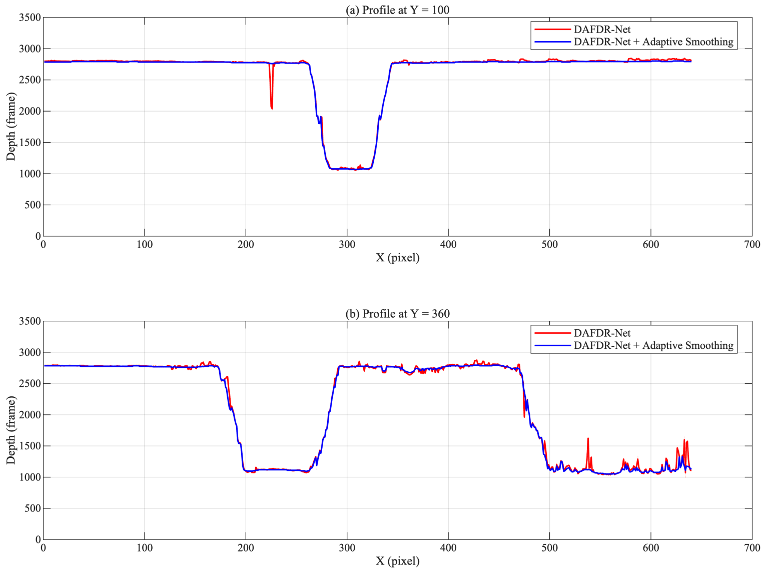

Figure S4. Depth profile comparison along two representative silicon-wafer profiles. Confidence-guided adaptive smoothing suppresses spikes while preserving mesa-edge transitions.

References

1. Nakagawa, Y.; Nayar, S. Shape from focus. *IEEE Trans. Pattern Anal. Mach. Intell.* **1994**, *16*, 824–831.
2. Xiong, Y.; Shafer, S.A. Depth from focusing and defocusing. In Proceedings of the IEEE Conference on Computer Vision and Pattern Recognition, New York, NY, USA, 15–17 June 1993; pp. 68–73.
3. Muhammad, M.S.; Choi, T.S. A novel method for shape from focus in microscopy using Bezier surface approximation. *Microsc. Res. Tech.* **2010**, *73*, 140–151.
4. Hu, J.; Shen, L.; Sun, G. Squeeze-and-excitation networks. In Proceedings of the IEEE Conference on Computer Vision and Pattern Recognition, Salt Lake City, UT, USA, 18–23 June 2018; pp. 7132–7141.
5. Pearson, R.K. Outliers in process modeling and identification. *IEEE Trans. Control Syst. Technol.* **2002**, *10*, 55–63. https://doi.org/10.1109/87.974338.
6. *ISO 25178-2:2012*; Geometrical Product Specifications (GPS)—Surface Texture: Areal—Part 2: Terms, Definitions and Surface Texture Parameters. International Organization for Standardization: Geneva, Switzerland, 2012.
